# Next-generation LTR-specific Tre-recombinase targets a majority of HIV-1 isolates

**DOI:** 10.1186/1471-2334-14-S2-O18

**Published:** 2014-05-23

**Authors:** Joachim Hauber, Janet Karpinski, Ilona Hauber, Jan Chemnitz, Helga Hofmann-Sieber, Claus-Henning Nagel, Niklas Beschorner, Carola Schäfer, Frank Buchholz

**Affiliations:** 1 Heinrich Pette Institute, Leibniz Institute for Experimental Virology, Hamburg, Germany; 2 University of Technology Dresden, University Hospital and Medical Faculty Carl Gustav Carus, Department of Medical Systems Biology, Dresden, Germany

## Introduction

HIV-1 integrates into the host chromosome and persists as a provirus flanked by long terminal repeats (LTR). To date, treatment regimens primarily target the virus enzymes, virus attachment or virus-cell fusion, but not the integrated provirus. Thus, current antiretroviral therapies (i.e. cART) cannot eradicate HIV-1, a fact that highlights the urgency of pursuing new strategies to find a cure for HIV/AIDS.

Previously, we engineered an experimental LTR-specific recombinase (Tre-recombinase) that can effectively excise integrated HIV-1 proviral DNA from infected human cell cultures (Sarkar et al. 2007 Science 316:1912). Subsequently, we demonstrated highly significant antiviral activity of this HIV-1 subtype A-specific Tre in humanized mice (Hauber et al. 2013 PLOS pathogens 9:e1003587). Broad clinical application, however, requires availability of a tre-recombinase that recognizes a majority of clinical HIV-1 isolates.

## Materials and methods

Here we report LTR target site identification as well as the engineering and functional analysis of a next-generation Tre-recombinase that recognizes the vast majority (e.g. >93% clade B and >80% clade A) of clinical HIV-1 isolates.

## Results

It is shown that the HIV-1 LTR harbours a conserved region that may serve as a universal tre recognition site for provirus excision. In fact, targeting this site by next-generation tre-recombinase demonstrates pronounced antiviral activity in the absence of cellular toxicity.

## Conclusion

The presented data suggest that next-generation Tre technology may be a valuable component of future antiretroviral therapies to reverse infection and thereby providing a cure for HIV/AIDS.

